# Systematic Review and Meta-analysis of the Role of Total Pancreatectomy as an Alternative to Pancreatoduodenectomy in Patients at High Risk for Postoperative Pancreatic Fistula: Is it a Justifiable Indication?

**DOI:** 10.1097/SLA.0000000000005895

**Published:** 2023-05-09

**Authors:** Thomas F. Stoop, Erik Bergquist, Rutger T. Theijse, Sebastian Hempel, Susan van Dieren, Ernesto Sparrelid, Marius Distler, Thilo Hackert, Marc G. Besselink, Marco Del Chiaro, Poya Ghorbani

**Affiliations:** * Division of Surgery, Department of Clinical Science, Intervention and Technology, Karolinska Institutet at Karolinska University Hospital, Stockholm, Sweden; † Amsterdam UMC, location University of Amsterdam, Department of Surgery; ‡ Cancer Center Amsterdam, Amsterdam, The Netherlands; § Division of Surgical Oncology, Department of Surgery, University of Colorado Anschutz Medical Campus, Aurora, CO; ∥ Department of Visceral, Thoracic and Vascular Surgery, University Hospital and Faculty of Medicine Carl Gustav Carus, Technische Universität Dresden, Dresden, Germany; ¶ Department of General, Visceral and Thoracic Surgery, University Hospital Hamburg-Eppendorf, Germany

**Keywords:** total pancreatectomy, pancreatoduodenectomy, pancreaticojejunostomy, high-risk, morbidity, mortality, quality of life, systematic review

## Abstract

**Objective:**

Examine the potential benefit of total pancreatectomy (TP) as an alternative to pancreatoduodenectomy (PD) in patients at high risk for postoperative pancreatic fistula (POPF).

**Summary Background Data:**

TP is mentioned as an alternative to PD in patients at high risk for POPF, but a systematic review is lacking.

**Methods:**

Systematic review and meta-analyses using Pubmed, Embase (Ovid), and Cochrane Library to identify studies published up to October 2022, comparing elective single-stage TP for any indication versus PD in patients at high risk for POPF. The primary endpoint was short-term mortality. Secondary endpoints were major morbidity (i.e., Clavien-Dindo grade ≥IIIa) on the short-term and quality of life.

**Results:**

After screening 1212 unique records, five studies with 707 patients (334 TP and 373 high-risk PD) met the eligibility criteria, comprising one randomized controlled trial and four observational studies. The 90-day mortality after TP and PD did not differ (6.3% *vs.* 6.2%; RR=1.04 [95%CI 0.56-1.93]). Major morbidity rate was lower after TP compared to PD (26.7% *vs.* 38.3%; RR=0.65 [95%CI 0.48-0.89]), but no significance was seen in matched/randomized studies (29.0% *vs.* 36.9%; RR = 0.73 [95%CI 0.48-1.10]). Two studies investigated quality of life (EORTC QLQ-C30) at a median of 30-52 months, demonstrating comparable global health status after TP and PD (77% [±15] *vs.* 76% [±20]; *P*=0.857).

**Conclusions:**

This systematic review and meta-analysis found no reduction in short-term mortality and major morbidity after TP as compared to PD in patients at high risk for POPF. However, if TP is used as a bail-out procedure, the comparable long-term quality of life is reassuring.

Postoperative pancreatic fistula (POPF) remains one of the most challenging complications after pancreatoduodenectomy (PD).^1^ Various preventive and therapeutic interventions have been studied with the aim of reducing the risk for and severity of POPF. However, most randomized trials in this field have been underpowered.^2^ Around one-third of patients with a high-risk pancreatico-enterostomy develop a clinically relevant (i.e., grade B/C) POPF that requires a change of management.^3^

Nowadays, POPF is often treated with minimally invasive management, possibly due to early recognition and a therapeutic step-up approach.^4–6^ Nevertheless, major morbidity and mortality of 72% and 18% have been reported in clinically relevant POPF^3,4^, whereby outcomes are even worse in up to 12% of patients^7^ who develop a POPF grade C with mortality rates reaching 56%.^8–10^

In recent years, there has been a renewed interest in total pancreatectomy (TP) as an alternative to PD to avoid severe POPF in case of very high-risk intraoperative conditions.^11–14^ The surgical outcome following TP has improved in particularly high-volume centers^15–18,^ whereby the subsequent metabolic insufficiencies have become more manageable with an acceptably reduced quality of life.^19–21^ In spite of that, this indication for TP still causes controversies. A systematic review of the increasing evidence on this topic is lacking.

The present systematic review and meta-analysis therefore aims to provide insight in the current evidence about the benefit of TP as an alternative to PD in patients at high risk for POPF.

## METHODS

This systematic review was performed in accordance with the Preferred Reporting Items for Systematic Reviews and Meta-Analyses (PRISMA) guidelines^22^ and was registered in the Prospective Register of Systematic Reviews (PROSPERO): CRD42022300700.^23^

### Study Population

**[P]** The study population included patients with any (suspected) pancreatic or periampullary pathology. **[I]** The intervention comprised preoperative scheduled TP or intraoperatively converted PD to TP for any indication, and **[C]** was compared with PD in patients at high risk for POPF. **[O]** The primary outcome of interest is mortality. Secondary outcomes include major morbidity (i.e., Clavien-Dindo grade ≥IIIa)^24^ and quality of life.^25^ Both major morbidity and mortality were measured during primary hospitalization or within 30- or 90-days after index surgery.

### Search Strategy

A systematic literature search was performed in Pubmed, Embase (Ovid), and the Cochrane Library to identify studies that were published from database inception to October 3^rd^, 2022. See Appendix 1, Supplemental Digital Content 1, http://links.lww.com/SLA/E587 for the search strategy. After removing duplicates, identified literature was independently screened by two authors (T.F.S. & E.B.) on title and abstract. Thereafter, the two authors screened the preliminary included articles in full text. See the in- and exclusion criteria below. In both screening phases, discrepancies were solved by consensus.

### Eligibility Criteria

Inclusion criteria comprised any comparative studies (i.e., PD in patients at high risk for POPF *versus* TP), reporting at least one of the primary or secondary endpoints. All definitions of high-risk pancreatico-enterostomy for POPF were allowed. Exclusion criteria included non-English literature, conference abstracts, letters to the editor, reviews, case series with less than 10 cases in at least one of the comparative arms, animal studies, and in which full text were not available.

### Critical Appraisal

Two authors (T.F.S. & E.B.) independently assessed the risk of bias from the included literature, whereby discrepancies were solved by consensus. Observational studies were assessed using the Newcastle Ottawa Scale^26^ and randomized controlled trials were scored by the Revised Cochrane Risk-of-Bias Tool for randomized trials.^27^ Studies with either a Newcastle Ottawa Scale score <7 points were classified as having a risk of bias.

### Data Synthesis and Statistical Analysis

Data analyses were performed with RStudio: Integrated Development Environment for R (software version 1.3.1093, Boston, MA). Meta-analyses were performed using the meta® and grid® packages.^28^ The Mantel-Haenszel random-effect model was used for the outcome measures major morbidity and mortality to calculate pooled risk ratio’s (RR) with their corresponding 95% confidence intervals (95%CI). Heterogeneity was assessed through the *I*^*2*^ metric and classified as ‘might not be important’ (0–40%), ‘may represent moderate heterogeneity’ (30–60%), ‘may represent substantial heterogeneity’ (50–90%), and ‘considerable heterogeneity’ (75–100%). Because of the few included studies, potential publication bias for the outcome measures were not inspected using funnel plots.

From included randomized controlled trials, the intention-to-treat analyses were used. If different included studies had overlapping cohorts, only the study with the highest level of evidence was used for the analyses. Studies using propensity score stratification were analysed as separate cohorts in the meta-analyses. Sensitivity analyses were performed to investigate the impact of studies with a risk of bias and the influence of unmatched/non-randomized studies and indications of TP on mortality and major morbidity.

Quality of life after TP and PD investigated with the EORTC QLQ-C30 (version 3.0) were compared, investigating the statistical difference (student *t-*test) and the clinical difference. The clinical difference was assessed with the methodology of Osoba and colleagues: <5% ‘no change’, 5–10% ‘little change’; 10–20% ‘moderate change’; and >20% ‘very much change’.^29^ The EORTC QLQ-C30 quality of life outcomes were pooled, wherefore medians with interquartile ranges had to be converted to means with standard deviations.^30,31^ Statistical significance was considered a two-tailed *P* value of <0.050.

## RESULTS

**FIGURE 1:**
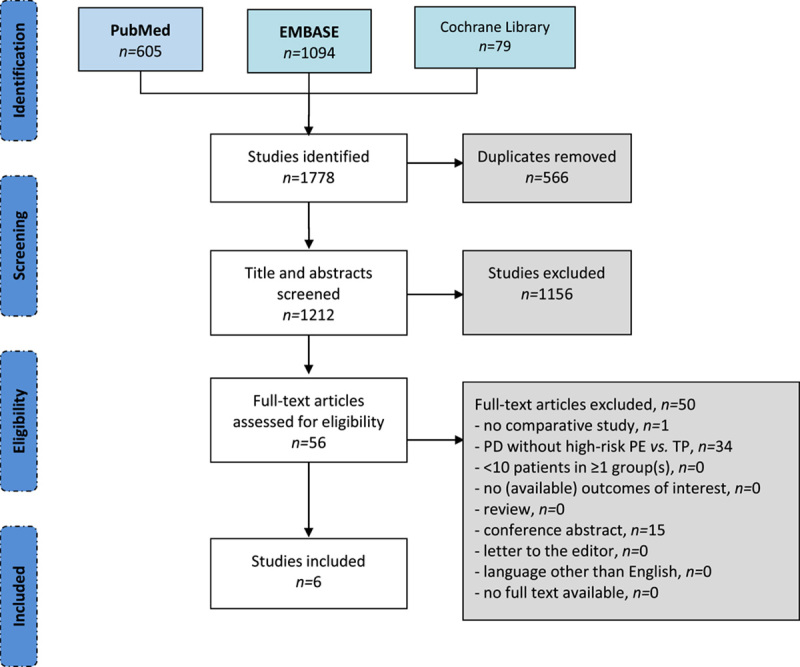
PRISMA flow chart. n indicates number of patients; PD, pancreatoduodenectomy; PE, pancreatico-enterostomy; TP, total pancreatectomy

The literature search identified 1212 unique studies of which six studies fulfilled the eligibility criteria.^32–37^ See Figure 1 for the PRISMA flow diagram. The six included studies comprised one multicenter randomized controlled trial,^33^ two single-center observational matched studies,^35,36^ and three single-center observational non-matched studies.^32,34,37^ One of the included studies was defined as having a high risk of bias.^34^ See Appendix 2, Supplemental Digital Content 2, http://links.lww.com/SLA/E588 for further details about the critical appraisal.

The studies originated from Italy (*n*=3),^32,33,37^ Germany (*n*=2),^34,35^ and Sweden (*n*=1)^36^ with an inclusion period ranging from 2008 until 2019. The inclusion criteria differed among the included studies as outlined in Appendix 3, Supplemental Digital Content 3, http://links.lww.com/SLA/E589. One observational study included all patients with periampullary cancer regardless of the risk for POPF whereby those patients who underwent a rescue pancreatectomy after PD were not included.^34^ Therefore, the *University Hospital and Faculty of Medicine Carl Gustav Carus (Dresden, Germany)* study group provided additional data, whereby the following patients were included: all patients with periampullary cancer who underwent a PD at high risk for POPF defined as an alternative Fistula Risk Score (aFRS) >20%^38^ or TP.

The observational single-center study from Capretti et al^32^ overlapped with the randomized controlled trial from Balzano and colleagues.^33^ Therefore, the study from Capretti and colleagues was excluded from the analyses,^32^ leaving five studies for the analyses.^33–37^

### Clinicopathological and Surgical Characteristics

The included studies comprised 707 patients, including 334 TP and 373 PD. Various definitions were used to define a high risk for POPF, as outlined in Table 1. In the 373 patients who underwent PD at high risk of POPF, a pancreatico-jejunostomy was performed in 91.2% (*n*=340). In the 334 patients who underwent TP, at least 8.4% of patients (*n*=28/334) underwent concomitant islet-autotransplantation (IAT). In the randomized controlled trial from Balzano and colleagues, 3 out of 31 patients (9.7%) randomized for the PD arm underwent an intraoperative conversion from PD to TP because of technical difficulties.^33^

**TABLE 1:** Baseline Characteristics

Study	Inclusion period	Design	TP (*n*)	Extended, *n* (%)	IAT, *n* (%)	Malignancy, *n* (%)	PD (*n*)	Extended, *n* (%)	Definition high-risk PE, *n* (%)	Type PE, (%)	Malignancy, *n* (%)
Capretti et al (2021)^32^ ^*^	2010-2019	R/S /M–	27	VR: 0 (0)	9 (15)	Unknown	35	VR: 2 (6)	FRS ≥7^†^ ^39^	PJ^‡^ (100)	Unknown
Hempel et al (2021)^34^ ^§^	2008-2017	R/S/M–	41	PVR: 23 (57) AR: 19 (46) MV: 16 (39)	Unknown	41 (100)	39	PVR: 25 (64) AR: 0 (0) MV: 0 (0)	aFRS >20%^38^	PJ (97) PG (3)	39 (100)
Luu et al (2021)^35^	2009-2018	R/S/M+	100	Unknown	Unknown	67 (67)	100	Unknown	Very soft parenchyma + duct <3 mm^¶^	PJ^∥^ (100)	67 (67)
Marchegiani et al (2021)^37^	2017-2019	R/S/M–	86^#^	VR: 33 (38)	Unknown	Unknown	101	VR: 5 (5)	aFRS >20%^38^	PJ (67) PG (32)	Unknown
Stoop et al (2022)^36^	2015-2017	R/S/M+									
		*Stratum 1:*	41	PVR: 30 (73) AR: 0 (0) MV: 17 (42)	0 (0)	35 (85)	18	PVR: 10 (56) AR: 0 (0) MV: 5 (28)	Soft parenchyma and/or duct ≤3 mm	PJ (100)	18 (100)
		*Stratum 2:*	24	PVR: 6 (25) AR: 0 (0) MV: 2 (8)	0 (0)	16 (67)	36	PVR: 5 (14) AR: 0 (0) MV: 0 (0)	Soft parenchyma and/or duct ≤3 mm	PJ (100)	17 (47)
		*Stratum 3:*	12	PVR: 0 (0) AR: 0 (0) MV: 0 (0)	0 (0)	9 (75)	48	PVR: 0 (0) AR: 0 (0) MV: 0 (0)	Soft parenchyma and/or duct ≤3 mm	PJ (100)	38 (79)
Balzano et al (2022)^33^ ^*^	2010-2019	P/M/RCT	30	Unknown	28 (93)	21 (70)	31	Unknown	Soft parenchyma and duct ≤3 mm	PJ (100)^∥^	24 (77)

* Overlapping cohorts.

† Patients in both the PD and TP group had an FRS ≥7. All TPs were initially scheduled as PD and intraoperatively converted because of pancreatic features and clinical condition.

‡ Two-layer end-to-side pancreaticojejunostomy.

§ The PD population differs from the primary publication, since that primary publication^34^ does not describe the outcomes from the patients who underwent a PD with high-risk PE separately. Therefore, the Hempel et al provided the data that meets the inclusion criteria of the present systematic review

∥ Double-layer end-to-side duct-to-mucosa pancreaticojejunostomy.

¶ Patients in both the PD and TP group had a very soft pancreatic remnant + pancreatic duct size <3 mm. TP was mainly performed because of the pancreatic remnant was found technically unsuitable for a safe anastomosis due to soft and friable pancreatic texture combined with small-sized pancreatic duct.

# All TPs were preoperatively scheduled as PD, but were intraoperatively converted to TP because of positive neck margin (49%), technical issues (27%), vascular resection/reconstruction (14%), or other reasons (10%; pancreatitis, bleeding, and iatrogenic splenic laceration).

aFRS indicates alternative fistula risk score; AR, arterial resection; mm, millimetres; IAT, islet-autotransplantation; M, multicentre; M-, no matching; M+, matching; n, number of patients; P, prospective study; PD, pancreatoduodenectomy; PE, pancreatico-enterostomy; PJ, pancreatojejunostomy; PVR, portomesenteric venous resection; R, retrospective study; RCT, randomized controlled trial; S, single-centre study; TP, total pancreatectomy, VR, vascular resection.

Only in two studies comprising 130 patients (38.9%) who underwent TP, all TPs were performed to avoid a POPF because of high-risk conditions.^33,35^ In contrast, the 86 patients who underwent TP (25.7%) from Marchegiani et al were all intraoperatively converted from PD to TP for any indication: positive pancreatic margin (*n*=42, 12.6%), technical issues (*n*=23, 6.9%), vascular resection/reconstruction (*n*=12, 3.6%), or other reason(s) (*n*=9, 2.7%).^37^ The remaining two studies included either all primary elective TPs regardless of the indication (23.1%)^36^ or all primary elective TPs for periampullary cancer (12.3%).^34^

In both the TP and PD group, the most common final histopathology was malignant periampullary or pancreatic disease (76% *vs.* 76%; RR=0.96 [95%CI 0.88–1.05]; *I*^*2*^=38%).^33–36^ These rates are calculated without the study from Marchegiani et al since they only described the presumed diagnosis and did not make a distinction between malignancy or benign/premalignant pathology.^37^ See Table 1 for the clinicopathological characteristics and procedural details.

### Surgical Outcomes

After PD, the rate of POPF grade B/C was 40% (95%CI, 30–52%; *I*^*2*^=72%),^33–37^ including a rescue pancreatectomy rate of 11% (95%CI, 7–18%; *I*^*2*^=34%).^33–36^

**FIGURE 2:**
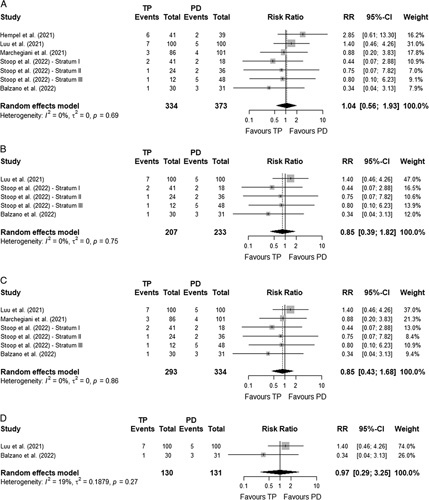
Meta-analysis – Mortality. A, Meta-analysis on mortality - Overall population. B, Meta-analysis on mortality - Matched/randomized controlled studies. C, Meta-analysis on mortality - Studies without high risk of bias. D, Meta-analysis on mortality - Studies with only TP performed because of a high risk for POPF.

The 90-day (or within hospitalization) mortality after TP and PD did not differ (6.3% *vs.* 6.2%; RR=1.04 [95%CI 0.56–1.93]; *I*^*2*^=0%),^33–37^ which remained in the sensitivity analyses on matched cohorts/randomized controlled trials (6.8% *vs.* 7.3%; RR=0.85 [95%CI 0.39–1.82]; *I*^*2*^=0%),^33,35,36^ studies with a low risk of bias (5.8% *vs.* 6.3%; RR=0.85 [95%CI 0.43–1.68]; *I*^*2*^=0%),^33,35–37^ and studies where TP was solely performed because of a high risk for POPF (6.2% *vs.* 6.1%; RR=0.97 [95%CI 0.29–3.25]; *I*^*2*^=19%).^33,35^ See Fig. 2A–D for the meta-analyses.

The reason for death was not described in two studies.^33,37^ In the remaining three series, 75.0% of the mortality after PD (*n*=12/16) was a consequence of POPF.^34–36^ This translates into a 5.0% (*n*=12/241) POPF-related mortality rate after PD.^34–36^ The cause of mortality after TP from these three studies^34–36^ were multi-organ failure by pneumonia followed by progressive respiratory failure (*n*=6/17, 35.3%), multi-organ failure due to intestinal ischemia (*n*=3/17, 17.6%), multi-organ failure due to hepatic failure after hepatic artery embolism (*n*=1/17, 5.9%), and multi-organ failure with an unknown cause (*n*=2/17, 11.8%). Other causes were myocardial infarction (*n*=1/17, 5.9%), cardiac arrest due to lung embolism (*n*=1/17, 5.9%), bile leakage followed by pneumonia and lung embolism (*n*=1/17, 5.9%), post-pancreatectomy hemorrhage followed by intestinal ischemia (*n*=1/17, 5.9%), or unknown cause (*n*=1/17, 5.9%).

**FIGURE 3:**
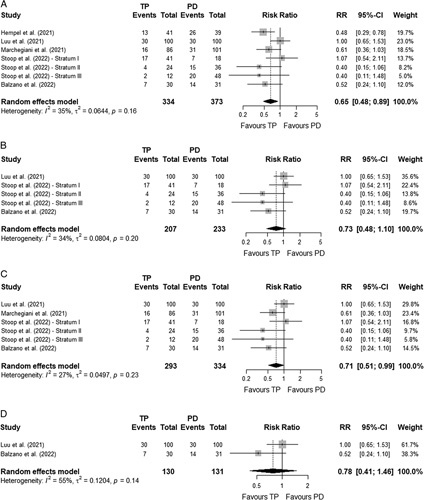
Meta-analysis – Major morbidity. A, Meta-analysis on major morbidity - Overall population. B, Meta-analysis on major morbidity - Matched/randomized controlled studies. C, Meta-analysis on major morbidity - Studies without high risk of bias. D, Meta-analysis on major morbidity - Studies with only TP performed because of a high risk for POPF.

Major morbidity was registered during 90 days (or within hospitalization)^33,35,37^ or just during primary hospitalization.^34,36^ The major morbidity rate was lower after TP compared to PD (26.7% *vs.* 38.3%; RR=0.65 [95%CI 0.48-0.89]; *I*^*2*^=35%),^33–37^ but the difference was no longer statistically significant when analysing only matched cohorts and randomized controlled trials (29.0% *vs.* 36.9%; RR=0.73 [95%CI 0.48-1.10]; *I*^*2*^=34%).^33,35,36^ See Fig. 3A–D for the meta-analyses.

The rate of post-pancreatectomy haemorrhage^40^ was lower after TP compared to PD (9.0% *vs.* 19.0%; RR=0.49 [95%CI 0.32–0.75]; *I*^*2*^=0%).^33–37^ The difference in favour of TP remained in the sensitivity analyses, except the analysis including studies in which TP was solely performed to avoid POPF (4.6% *vs.* 14.5%; RR=0.34 [95%CI 0.10–1.17]; *I*^*2*^=44%).^33,35^ See Appendix 4, Supplemental Digital Content 4, http://links.lww.com/SLA/E590 for the meta-analyses.

The rate of bile leakage after TP and PD did not differ (6.0% *vs.* 8.8%; RR=0.80 [95%CI 0.44–1.47]; *I*^*2*^=0%),^33–37^ neither in the sensitivity analyses. See Appendix 5, Supplemental Digital Content 5, http://links.lww.com/SLA/E591 for the meta-analyses. Here, two of the five studies used the 2007 International Study Group for Liver Surgery (ISGLS) definition.^41^

### Quality of life

Two studies cross-sectionally investigated the postoperative quality of life,^36,37^ using the EORTC QLQ-C30 (version 3.0) questionnaire.^42^ The median postoperative moment of quality of life-assessment ranged from 30 to 52 months. The global health status after TP and PD were similar (77% [±15] *vs.* 76% [±20]; *P*=0.857; no clinically significant difference), as well as all different functioning parameters. Patients after TP suffered more (often) from insomnia (30% [±32] *vs.* 18% [±27]; *P*=0.012; moderate clinical significance). In contrast, appetite loss (5% [±13] *vs.* 11% [±23]; *P*=0.038; little clinical significance) and constipation (5% [±13] *vs.* 11% [±23]; *P*=0.025; little clinical significance) were worse after PD. See Table 2 for all quality of life-outcomes.

**TABLE 2:** Quality of life – QLQ-C30 (version 3.0)

	Marchegiani et al^37^		Stoop et al^36^						Overall				
			Stratum 1		Stratum 2		Stratum 3				*P*-value	Clinical significance^29^	
	TP (*n*=33)	PD (*n*=62)	TP (*n*=13)	PD (*n*=5)	TP (*n*=16)	PD (*n*=18)	TP (*n*=10)	PD (*n*=20)	TP (*n*=72)	PD (*n*=105)		Δ	
Global health status	79 (15)	78 (19)	76 (12)	67 (20)	73 (15)	74 (26)	77 (16)	77 (17)	77 (15)	76 (20)	0.857	+ 0.5	No change
Functioning													
Physical functioning	88 (12)	85 (20)	79 (23)	77 (14)	76 (11)	81 (20)	81 (14)	86 (22)	83 (15)	84 (20)	0.584	− 1.5	No change
Role functioning	86 (20)	85 (25)	69 (29)	77 (22)	77 (22)	72 (32)	75 (26)	83 (24)	80 (23)	82 (26)	0.552	− 2.3	No change
Emotional functioning	82 (20)	80 (22)	91 (9)	77 (18)	88 (17)	82 (21)	89 (13)	87 (20)	86 (17)	82 (21)	0.160	+ 4.2	No change
Cognitive functioning	90 (13)	86 (20)	88 (13)	97 (7)	79 (16)	82 (18)	83 (26)	89 (22)	87 (15)	87 (19)	0.971	− 0.1	No change
Social functioning	88 (18)	85 (25)	64 (22)	80 (30)	75 (24)	70 (35)	82 (28)	87 (18)	80 (22)	83 (26)	0.424	− 3.0	No change
Symptoms													
Fatigue	16 (19)	19 (23)	30 (28)	24 (9)	39 (15)	30 (31)	32 (15)	25 (21)	26 (20)	22 (24)	0.255	+ 3.9	No change
Nausea & vomiting	5 (14)	4 (9)	5 (11)	13 (22)	8 (18)	13 (32)	5 (11)	11 (17)	6 (14)	7 (17)	0.439	− 1.9	No change
Pain	12 (19)	8 (17)	14 (23)	13 (14)	22 (19)	24 (31)	15 (21)	19 (22)	15 (20)	13 (21)	0.605	+ 1.6	No change
Dyspnoea	4 (14)	6 (17)	23 (32)	40 (28)	23 (20)	24 (23)^*^	27 (27)	12 (16)	15 (21)	12 (18)	0.324	+ 3.0	No change
Insomnia	30 (29)	22 (30)	23 (29)	13 (18)	31 (33)	15 ((23)	33 (42)	12 (20)	30 (32)	18 (27)	**0.012**	+ 11.2	Moderate change
Appetite loss	5 (12)	9 (20)	0 (0)	13 (18)	6 (13)	13 (28)	7 (21)	16 (28)^*^	5 (13)	11 (23)	**0.038**	− 6.3	Little change
Constipation	5 (12)	9 (21)	0 (0)	20 (30)	4 (11)	13 (28)	10 (23)	15 (23)	5 (13)	11 (23)	**0.025**	− 6.8	Little change
Diarrhoea	18 (26)	24 (28)	38 (33)	13 (18)	31 (33)	22 (30)	30 (33)	18 (23)	26 (30)	22 (27)	0.338	+ 4.2	No change
Financial difficulties	10 (21)	10 (22)	5 (13)	0 (0)	15 (27)	9 (25)	0 (0)	12 (27)	9 (20)	9 (23)	0.761	−1.0	No change

Bold values are statistically significant.

All values are on a scale from 0 to 100%, expressed in means with standard deviation (SD), unless expressed differently. High scores of global health status and functioning indicate good quality of life, whereas high symptom scores suggest poor quality of life.

* n=1 missing data.

PD indicates pancreatoduodenectomy; TP, total pancreatectomy.

## DISCUSSION

This first systematic review and meta-analysis investigating the role of TP as an alternative to PD in patients at high risk for POPF included six studies with 711 patients (334 TP and 373 PD) and demonstrated similar 90-day mortality rates (6% *vs.* 6%), whereas major morbidity rates were in favor of TP (27% *vs.* 38%). This difference in major morbidity was no longer significant when analysing only matched/randomized controlled studies (29% *vs.* 37%). The quality of life after TP and PD in the middle-/long-term were comparable, based on data from two observational studies.

In the present study, the pooled rate of POPF grade B/C was 40%, which is higher than the 23% rate in patients who underwent PD in the presence of the International Study Group of Pancreatic Surgery (ISGPS) type D high-risk features (i.e., soft pancreatic tissue + main pancreatic duct size ≤3 mm).^43,44^ This also explains the high rate of rescue pancreatectomies (11%) in comparison to the median of 2% in 6186 patients who underwent PD in high-volume centers.^7^ A worldwide variety exists regarding prevention and management strategies of POPF.^45^ This is illustrated by the wide range of POPF grade C incidences among centers (0% to 12% in even high-volume centers),^7^ possibly influenced by different strategies on minimally invasive versus surgical management of clinically relevant POPF.^8,10^ The PORSCH trial showed that an algorithm-guided early detection of POPF and minimally invasive management improved clinical outcomes following pancreatic surgery regardless of hospital volume, resulting in a 50% reduction of 90-day mortality (5% to 3%) after PD.^5^ These findings have further challenged the role of TP in avoiding POPF.

The recently published PAN-IT trial is the only level 1 evidence in this systematic review, randomizing patients intraoperatively for PD *versus* TP-IAT in the presence of high-risk conditions (i.e., ISGPS type D).^33^ Since the primary outcome (i.e., 90-day morbidity) was lower after TP and the absence of serious hypoglycaemic events, the authors concluded that TP-IAT might become the standard treatment in candidates for PD when a high risk of POPF is predicted.^33^ The PAN-IT trial has several major limitations regarding the primary endpoint, lack of patient-reported outcome measures, and limited quality of diabetes control.^46^ Therefore, the authors’ conclusion could be interpreted as ‘too strong’. The meta-analysis in this systematic review, including only matched/randomized studies (including the PAN-IT trial), revealed similar major morbidity (29% *vs.* 37%) and mortality (7% *vs.* 7%) rates after TP and PD.

It remains questionable whether an eight percent point reduction of (major) morbidity without benefits in 90-day mortality justifies a lifelong apancreatic state. The endocrine and exocrine insufficiencies after TP are more manageable nowadays^19–21,47,48^ with acceptably reduced quality of life.^18,21,36,37,49^ The quality of life after TP is even reported to be comparable to patients after PD, as demonstrated by the findings in this systematic review. Nevertheless, the ‘similarities’ in quality of life between TP and PD need to be interpreted with caution. The cross-sectional assessment was mostly executed on the middle- and long-term. Therefore, the first month(s) and year(s) after surgery remain relatively unexplored, while patients are adapting to their insufficiencies during that period of time. Furthermore, a significant group of patients will not reach that middle- or long-term due to disease recurrence,^50–52^ although patients with a high risk for POPF often have non-pancreatic periampullary adenocarcinoma or benign/premalignant pancreatic pathology associated with a better prognosis.

The recently started TETRIS trial (NCT05212350) randomizes patients with a (very) high risk for POPF to either TP or PD, with major morbidity as a primary endpoint.^53^ Furthermore, the recently started single-arm TPIAT-01 trial (NCT05116072) investigates the efficacy and safety of TP-IAT for resectable pancreatic head cancer at high risk for developing POPF, aiming to improve the completion rate of adjuvant chemotherapy.^54^ Although it is established that POPF is associated with delay or renouncement of adjuvant chemotherapy,^55–57^ using this as an indication for TP(-IAT) seems too radical in an era where patients with resectable pancreatic adenocarcinoma are increasingly treated with neoadjuvant therapy,^58,59^ although the level 1 evidence remains conflicting.^60,61^ To investigate the definite role of TP as an alternative for PD with a high-risk pancreatico-enterostomy, mortality, and patient-reported outcome^62^ measures should be considered as more/the only appropriate primary endpoints. Reducing morbidity alone as a rationale to perform TP as an alternative to PD is no longer sufficient,^46^ since morbidity and mortality due to POPF can be strongly reduced by early detection and minimally invasive management in a systematic, algorithm-guided manner.^5^

Regardless of the ambiguity concerning the role of TP for this indication, TP might be an option in highly selected patients considering the substantial but non-significant lower 90-day mortality after TP-IAT in the PAN-IT trial.^33^ After all, the included, PAN-IT trial was the only powered study, but without mortality or patient-reported outcome as primary endpoints. For instance, TP might be considered in pre-existent vulnerable patients with severe systemic diseases who are prone for ‘failure to rescue’.^63^ After all, predicting severe POPF remains highly challenging^64^ and failure to rescue resulting in a rescue pancreatectomy is associated with high mortality reaching 56%.^8–10^ To avoid such life-threatening complications in pre-existent vulnerable patients, TP might be an alternative. Probably the most accepted scenario in which TP might be considered to avoid POPF-related complications is arterial reconstruction during PD, because of the risk for erosive life-threatening post-pancreatectomy hemorrhages.^11,65^ On the other hand, the protective effect of TP in the case of arterial resections is challenged by others.^66^ It is concerning that 75% of mortality after high-risk PD in this systematic review is related to POPF, whereas 25% of patients died by other causes. Future studies should address this and aim to reduce the rate of POPF and subsequent other complications and mortality in these patients.

In the above-mentioned scenarios, surgeons should strive for case-specific preoperative shared decision-making, balancing the pros and cons, including the patients’ capability to learn how to manage the endocrine and exocrine insufficiencies.^67^ If TP is deemed necessary as a bail-out option, TP-IAT^68,69^ or the bi-hormonal artificial pancreas could be considered, reducing the severeness and burden of the endocrine insufficiencies.^70^ Based on these arguments, one might argue that the indication for TP – in the presence of high-risk conditions for a pancreatico-enterostomy – are not generalizable to all patients with high-risk features. Therefore, performing a randomized controlled trial could even be questioned.

The findings of the present systematic review and meta-analysis have to be interpreted in the light of several limitations. First, only five studies were included, of which four retrospective observational series (including only two matched studies) and one randomized controlled trial whereby the latter was powered on overall complications as the primary endpoint. This hampers the capability to draw strong conclusions, and even more in sensitivity analyses. Second, various definitions of high risk for POPF were used in the included studies, leading to a heterogeneous population. Third, only two studies included solely TP performed to avoid POPF. Fourth, all included studies come from high-income countries with assumingly high standards of healthcare, including diabetes care. Taking this into consideration, TP should be performed with even more reluctance in countries with less developed healthcare systems to avoid diabetes-related morbidity and mortality. Fifth, the limited available literature on this topic reduced the strength of the present meta-analyses. Sixth, potential differences in mitigation strategies among centers could have influenced the surgical outcome in de PD group. Nevertheless, this is the first systematic review and meta-analysis that collected and analysed the expanding literature on the role of TP as an alternative to PD in patients at high risk for POPF.

## CONCLUSION

This systematic review and meta-analysis did not find a reduction in short-term mortality and major morbidity after TP as compared to PD in patients at high risk for POPF. This should be seen in light of the inevitable endocrine and exocrine insufficiencies after TP(-IAT). The definite value of TP needs to be established in adequately designed randomized trials focussing on mortality and quality of life. If TP is considered intraoperatively as a bail-out option, for instance, when technical difficulties in constructing a pancreatico-enterostomy arises in vulnerable patients or in cases of arterial reconstruction, surgeons should not be overly reluctant to perform TP given the reassuring comparable long-term quality of life after TP and PD.

## Supplementary Material
