# Supplementary material for: Systematic Review and Meta-analysis of the Role of Total Pancreatectomy as an Alternative to Pancreatoduodenectomy in Patients at High Risk for Postoperative Pancreatic Fistula: Is it a Justifiable Indication?
Source: Ann Surg. 2023 May 9;278(4):e702–11. doi: 10.1097/SLA.0000000000005895 (PMC10481933; doi:10.1097/SLA.0000000000005895)
Supplement: Supplementary file 1 [file sla-278-e702-s001.docx]

| **APPENDIX 1.** Search strategy | | |
| --- | --- | --- |
| **Database** | **Syntax** | **Hits** |
| **PubMed** | (total pancreatectom*[tiab]) AND (pancreatoduodenectom*[tiab] OR pancreaticoduodenectom* [tiab] OR pancreatic head resection*[tiab]) | **605** |
| **Embase (Ovid)** | (total pancreatectom* and (pancreatoduodenectom* or pancreaticoduodenectom* or pancreatic head resection*)).ti,ab,kf. | **1094** |
| **The Cochrane Library** | ((total pancreatectom*) AND (pancreatoduodenectom* OR pancreaticoduodenectom*)):ti,ab,kw | **79** |
| **Overall** | | **1778** |
