# Supplementary material for: Systematic Review and Meta-analysis of the Role of Total Pancreatectomy as an Alternative to Pancreatoduodenectomy in Patients at High Risk for Postoperative Pancreatic Fistula: Is it a Justifiable Indication?
Source: Ann Surg. 2023 May 9;278(4):e702–11. doi: 10.1097/SLA.0000000000005895 (PMC10481933; doi:10.1097/SLA.0000000000005895)
Supplement: Supplementary file 2 [file sla-278-e702-s002.docx]

| **APPENDIX 2a.** Methodological quality assessment of included observational studies | | | | | | | | | | | | |
| --- | --- | --- | --- | --- | --- | --- | --- | --- | --- | --- | --- | --- |
|  | **Selection** | | | |  | **Comparability** |  | **Outcomes** | | |  | **Total** |
| **Study** | **Representativeness** | **Selection of the non-exposed** | **Ascertainment of exposure** | **Conflict of interest** |  | **On the basis of the design of analysis** |  | **Assessment** | **Length of FU** | **Adequacy of FU ^#^** |  |  |
| **Capretti et al. (2021)^32^** | ● | ● | ● | ● |  | ● ○ |  | ● | ● | ● |  | **8** |
| **Hempel et al. (2021)^34^** | ○ | ● | ● | ● |  | ○ ○ |  | ● | ● | ● |  | **6** |
| **Luu et al. (2021)^35^** | ● | ● | ● | ● |  | ● ● |  | ● | ● | ● |  | **9** |
| **Marchegiani et al. (2021)^37^** | ● | ● | ● | ● |  | ● ○ |  | ● | ● | ● |  | **8** |
| **Stoop et al. (2022)^36^** | ● | ● | ● * | ● |  | ● ● |  | ● * | ● | ● |  | **9** |
| Risk of bias assessment using the Newcastle Ottawa Scale.  *FU,* follow-up  ●, meets the criteria from the Newcastle Ottawa Scale  ○, does NOT meet the criteria from the Newcastle Ottawa Scale  #, adequacy of follow-up is assessed for the primary outcome of this systematic review (i.e., mortality)  *, authors are aware of the methodology, but these criteria were not clearly described in the original manuscript | | | | | | | | | | | | |

| **APPENDIX 2b.** Methodological quality assessment of included randomized controlled trials | |
| --- | --- |
| **Criteria** | **Balzano et al. (2022)^33^** |
| Domain 1: Risk of bias arising from the randomization process | Low risk |
| Domain 2: Risk of bias due to deviations from the intended interventions | Low risk |
| Domain 3: Risk of bias due to missing outcome data | Low risk |
| Domain 4: Risk of bias in measurement of the outcome | Low risk |
| Domain 5: Risk of bias in selection of reported result | Low risk |
| **Overall risk-of-bias judgement** | **Low risk** |
| Risk of bias assessment using the Revised Cochrane Risk-of-Bias Tool for randomized trials. | |
