# Supplementary material for: Systematic Review and Meta-analysis of the Role of Total Pancreatectomy as an Alternative to Pancreatoduodenectomy in Patients at High Risk for Postoperative Pancreatic Fistula: Is it a Justifiable Indication?
Source: Ann Surg. 2023 May 9;278(4):e702–11. doi: 10.1097/SLA.0000000000005895 (PMC10481933; doi:10.1097/SLA.0000000000005895)
Supplement: Supplementary file 3 [file sla-278-e702-s003.docx]

| **APPENDIX 3.** In- and exclusion criteria per study | | | | |
| --- | --- | --- | --- | --- |
|  | **Pancreatoduodenectomy** | | **Total pancreatectomy** | |
| **Study** | **Inclusion criteria** | **Exclusion criteria** | **Inclusion criteria** | **Exclusion criteria** |
| **Capretti et al. (2021)^32^** | - PD for any indication | - FRS <7 points | - Intraoperatively converted PD to TP based on pancreatic features and patient’s clinical condition | - FRS <7 points  - Completion pancreatectomy for oncological reason |
| **Hempel et al. (2021)^34^** | - PD for periampullary cancer | - aFRS ≤20% | - TP for periampullary cancer | - None |
| **Luu et al. (2021)^35^** | - PD for any indication  - Very soft pancreatic remnant + pancreatic duct size <3mm | - None | - Primary elective TP for high-risk pancreatic remnant  - Very soft pancreatic remnant + pancreatic duct size <3mm | - None |
| **Marchegiani et al. (2021)^37^** | - Open PD for any indication | - aFRS ≤20% | - Intraoperatively converted PD to TP for any indication | - None |
| **Stoop et al. (2022)^36^** | - Age ≥18 years  - PD with high-risk PJ (i.e., soft pancreatic tissue and/or pancreatic duct size ≤3mm | - Concomitant arterial resection  - Non-elective pancreatectomy  - History of pancreatic surgery  - Missing data on variables using for propensity score matching | - Age ≥18 years | - Concomitant arterial resection  - Non-elective pancreatectomy  - History of pancreatic surgery  - Missing data on variables using for propensity score matching |
| **Balzano et al. (2021)^33^** | - Age ≥18 years  - Soft pancreas + pancreatic duct size  ≤3mm  - Fasting glycemia <126 mg/dl without glucose lowering medication | - Multifocal neoplasia (including multifocal IPMN and/or main duct dilatation affecting distal pancreas)  - Positive resection margin at frozen section examination | - Age ≥18 years  - Soft pancreas + pancreatic duct size ≤3mm  - Fasting glycemia <126 mg/dl without glucose lowering medication | - Multifocal neoplasia (including multifocal IPMN and/or main duct dilatation affecting distal pancreas)  - Positive resection margin at frozen section examination |
| *PD,* pancreatoduodenectomy; *PE*, pancreatico-enterostomy; *TP,* total pancreatectomy; *FRS,* fistula risk score; *aFRS,* alternative Fistula Risk Score; *mm,* millimetres; *PJ,* pancreatico-jejunostomy; *IPMN,* intraductal papillary mucinous neoplasm; *mg,* milligram; *dl,* decilitre. | | | | |
