# Supplementary material for: Systematic Review and Meta-analysis of the Role of Total Pancreatectomy as an Alternative to Pancreatoduodenectomy in Patients at High Risk for Postoperative Pancreatic Fistula: Is it a Justifiable Indication?
Source: Ann Surg. 2023 May 9;278(4):e702–11. doi: 10.1097/SLA.0000000000005895 (PMC10481933; doi:10.1097/SLA.0000000000005895)
Supplement: Supplementary file 4 [file sla-278-e702-s004.docx]

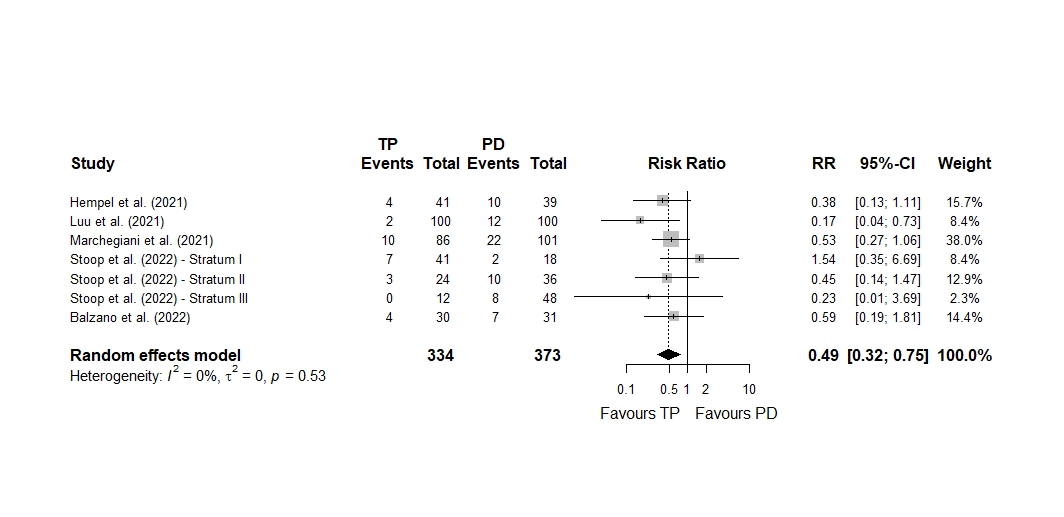
**Appendix 4a.** Meta-analysis on post-pancreatectomy hemorrhage **–** Overall population

**
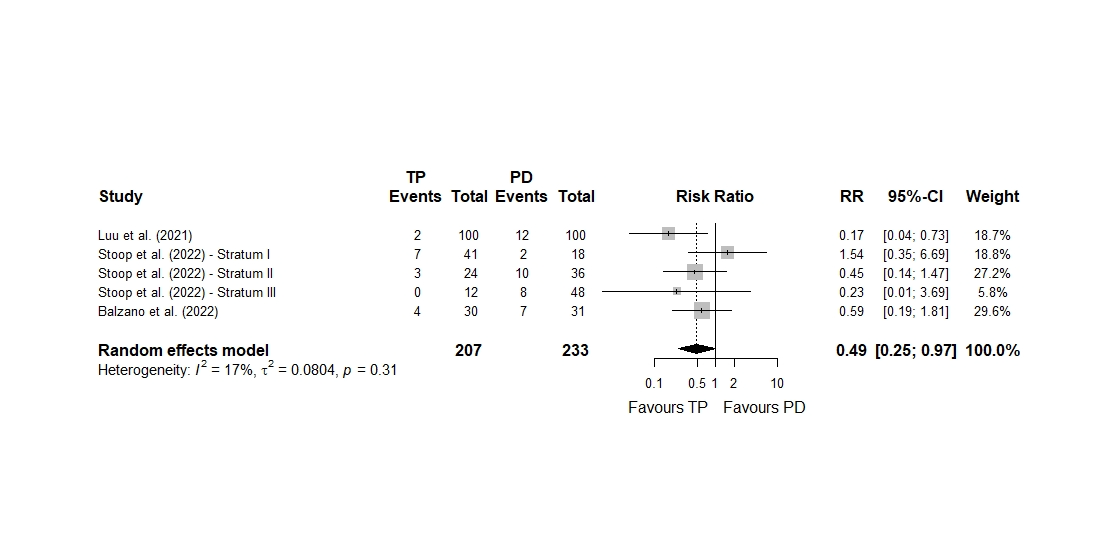
Appendix 4b.** Meta-analysis on post-pancreatectomy hemorrhage **–** Matched/randomized controlled studies

**
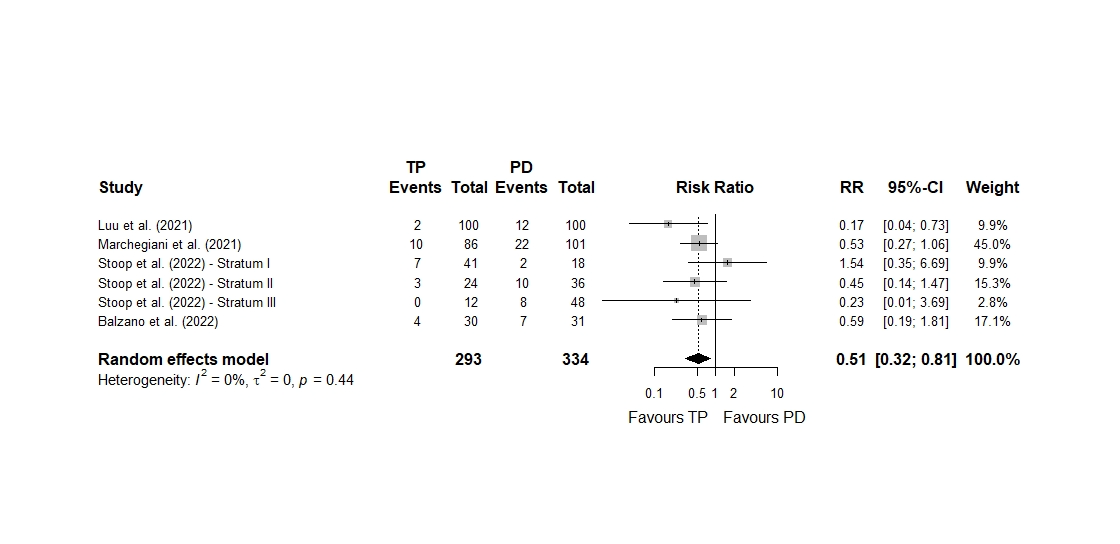
 Appendix 4c.** Meta-analysis on post-pancreatectomy hemorrhage **–** Studies without high risk of bias

**Appendix 4d.** Meta-analysis on post-pancreatectomy hemorrhage **–** Studies with only TP performed because of a high risk for POPF

**
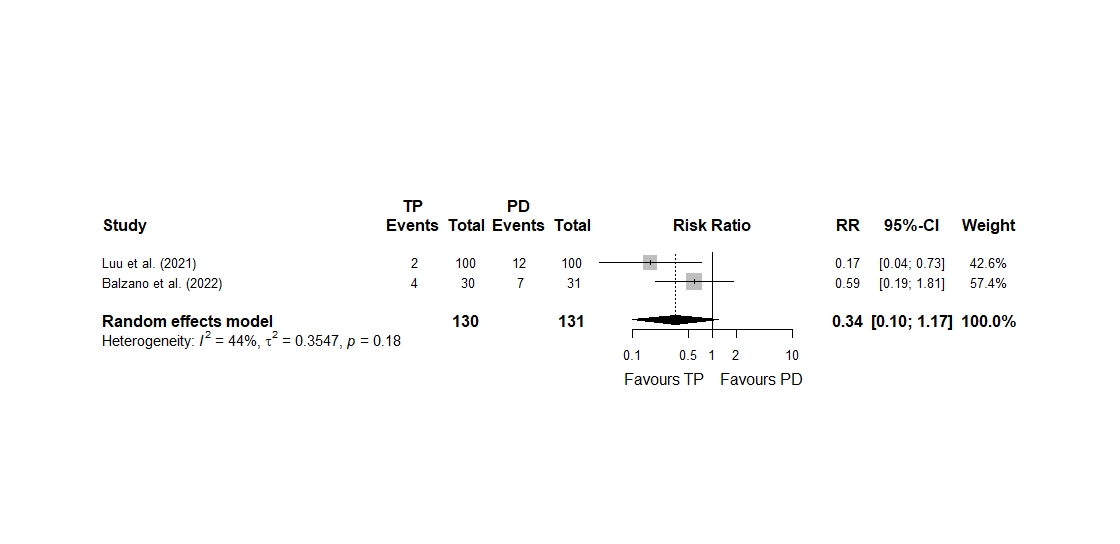
**

*TP,* total pancreatectomy; *PD,* pancreatoduodenectomy; *POPF,* postoperative pancreatic fistula; *RR,* relative risk; *95%-CI,* 95% confidence interval. **All included studies used the 2007 ISGPS definition for post-pancreatectomy hemorrhage. Events from Luu *et al.* and Balzano *et al.* concern post-pancreatectomy grade A-B-C. The events from Hempel *et al.*, Marchegiani *et al*. and Stoop *et al.* concerns grade B-C.**
