# Supplementary material for: Systematic Review and Meta-analysis of the Role of Total Pancreatectomy as an Alternative to Pancreatoduodenectomy in Patients at High Risk for Postoperative Pancreatic Fistula: Is it a Justifiable Indication?
Source: Ann Surg. 2023 May 9;278(4):e702–11. doi: 10.1097/SLA.0000000000005895 (PMC10481933; doi:10.1097/SLA.0000000000005895)
Supplement: Supplementary file 5 [file sla-278-e702-s005.docx]

**
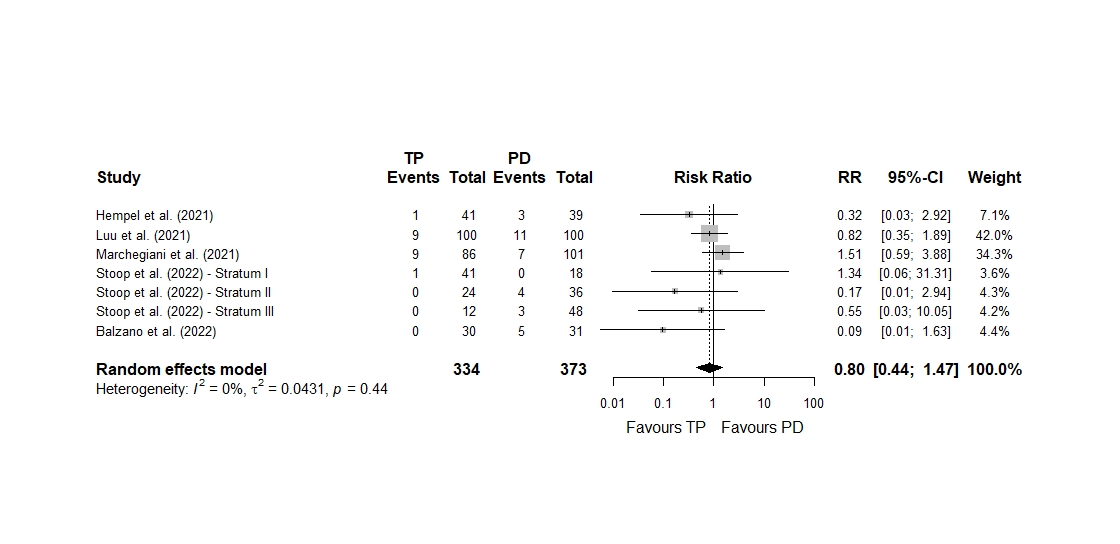
APPENDIX 5a.** Meta-analysis on bile leakage **–** Overall population

**
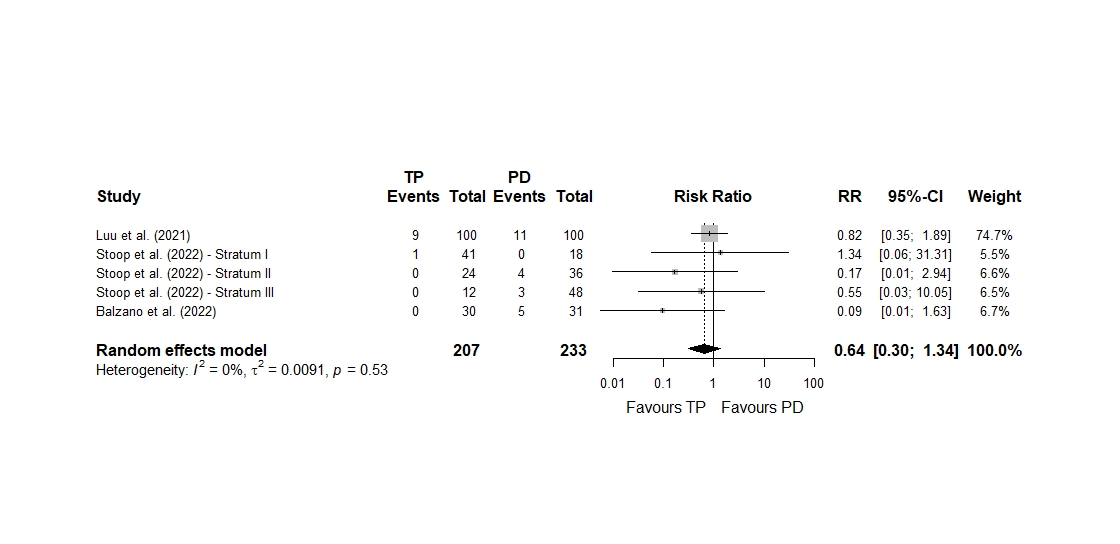
APPENDIX 5b.** Meta-analysis on bile leakage **–** Matched/randomized controlled studies

**
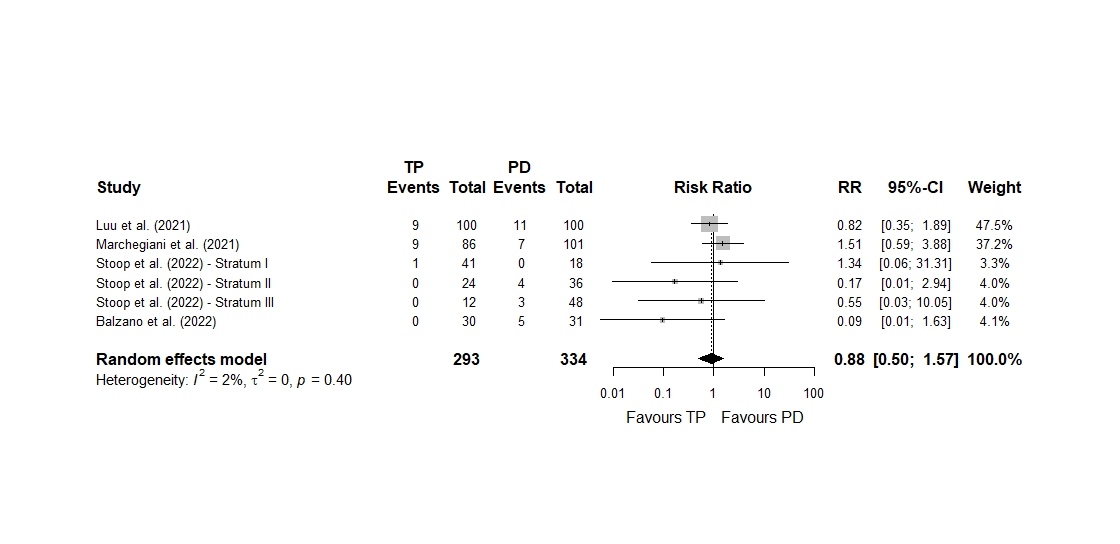
APPENDIX 5c.** Meta-analysis on bile leakage **–** Studies without high risk of bias

**APPENDIX 5d.** Meta-analysis on bile leakage **–** Studies with only TP performed because of a high risk for POPF


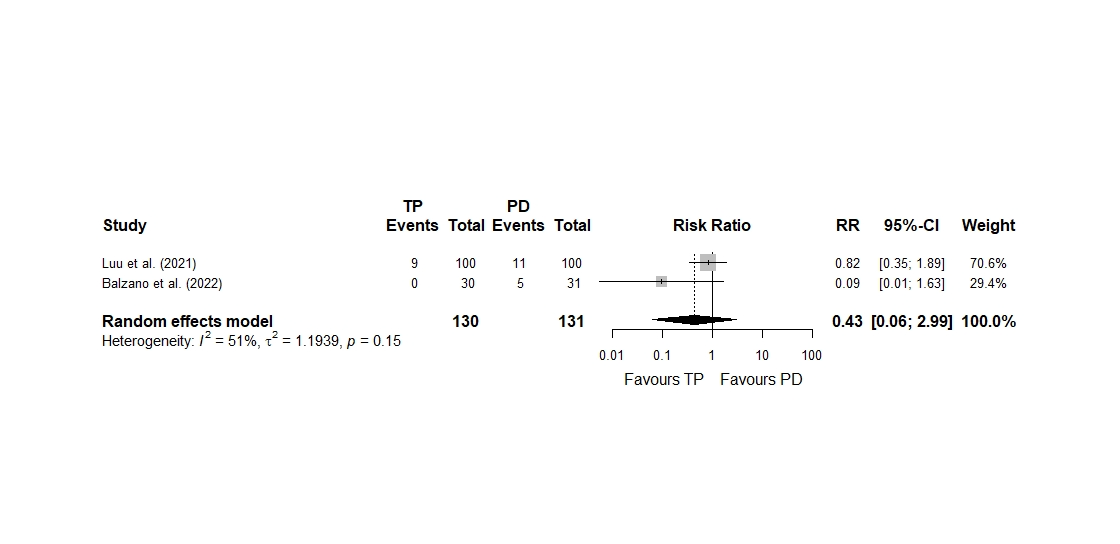


*TP,* total pancreatectomy; *PD,* pancreatoduodenectomy; *POPF,* postoperative pancreatic fistula; *RR,* relative risk; *95%-CI,* 95% confidence interval. **Hempel *et al.* and Stoop *et al.* used the 2007 ISGLS definition for bile leakage whereby events were defined as grade B-C. The other studies did not use a classification, therefore present bile leakage as yes versus no.**
